# Combinatorial gene therapy renders increased survival in cirrhotic rats

**DOI:** 10.1186/1423-0127-17-42

**Published:** 2010-05-28

**Authors:** Francisco J Gálvez-Gastélum, Aida A Segura-Flores, María D Senties-Gomez, Jose F Muñoz-Valle, Juan S Armendáriz-Borunda

**Affiliations:** 1 Institute for Molecular Biology in Medicine and Gene Therapy, University of Guadalajara, Department of Molecular Biology and Genomics, Sierra Mojada St. #950, Guadalajara (44280), Mexico; 2 O.P.D. Hospital Civil de Guadalajara, Sierra Mojada St. #950, Guadalajara (44280), Mexico

## Abstract

**Background:**

Liver fibrosis ranks as the second cause of death in México's productive-age population. This pathology is characterized by acummulation of fibrillar proteins in hepatic parenchyma causing synthetic and metabolic disfunction. Remotion of excessive fibrous proteins might result in benefit for subjects increasing survival index. The goal of this work was to find whether the already known therapeutical effect of human urokinase Plasminogen Activator and human Matrix Metalloprotease 8 extends survival index in cirrhotic animals.

**Methods:**

Wistar rats (80 g) underwent chronic intoxication with CCl_4_: mineral oil for 8 weeks. Cirrhotic animals were injected with a combined dose of Ad-delta-huPA plus Ad-MMP8 (3 × 10^11 ^and 1.5 × 10^11 ^vp/Kg, respectively) or with Ad-beta-Gal (4.5 × 10^11^) and were killed after 2, 4, 6, 8 and 10 days. Then, liver and serum were collected. An additional set of cirrhotic animals injected with combined gene therapy was also monitored for their probability of survival.

**Results:**

Only the cirrhotic animals treated with therapeutical genes (Ad-delta-huPA+Ad-MMP-8) showed improvement in liver fibrosis. These results correlated with hydroxyproline determinations. A significant decrement in alpha-SMA and TGF-beta1 gene expression was also observed. Cirrhotic rats treated with Ad-delta-huPA plus Ad-MMP8 had a higher probability of survival at 60 days with respect to Ad-beta-Gal-injected animals.

**Conclusion:**

A single administration of Ad-delta-huPA plus Ad-MMP-8 is efficient to induce fibrosis regression and increase survival in experimental liver fibrosis.

## Background

Advanced liver fibrosis and/or cirrhosis, represent a worldwide health problem. In México, represent the 2^nd ^cause of dead in productive-age population [1]. This pathology is consequence of a sustained chronic hepatic injury by a variety of causes including viral, chronic alcohol abuse and cholestasis induced by prolonged biliary obstruction [2,3].

Multiple factors influencing survival of patients with hepatic cirrhosis are invoked. Etiology is the principal determinant, though, factors as age, life style and the presence of complications at moment of diagnosis (ascitis, ictericia, encephalopathy, variceal haemorrhage and others) impact in the survival of these patients [3].

Accumulation of extracellular matrix (ECM) proteins distorts the hepatic architecture by forming a fibrous scar, and the subsequent development of nodules of regenerating hepatocytes defines cirrhosis. Cirrhosis produces hepatocellular dysfunction and increased intrahepatic resistance to blood flow, which result in hepatic insufficiency and portal hypertension [2,4].

Currently, therapeutic repertoire for liver fibrosis and cirrhosis treatment is limited. Broadly, treatment falls into two categories; removal of the underlying injurious stimulus (where possible), such as viral eradication in hepatitis B- and C-mediated liver disease, and liver transplantation, though with existing disadvantage [4,5].

Central to fibrogenesis and the scarring of organs is the activation of tissue fibroblasts into ECM-secreting myofibroblasts. Within the liver, the main effector cells of fibrosis are activated-hepatic stellate cells (aHSC), that express (among other pro-fibrogenic molecules) TGF-β and secrete fibrillar collagens, resulting in the deposition of fibrotic matrix. HSC also express TIMP with the result that ECM-degrading metalloproteinase activity is inhibited. This alters the balance and renders ECM accumulation [2,4,6].

Matrix metalloproteinases (MMPs) are a family of zinc-dependent proteolytic enzymes which comprise 22 different members. These can degrade virtually all the constituents of the ECM [7,8]. Although all of them exhibit a broad substrate spectrum, they are divided based on their main substrate into collagenases, gelatinases, stromelysins, matrilysins, metalloelastases, membrane-type MMPs (MT-MMPs), and others [8]. In particular MMP-8 is a neutrophil collagenase that avidly degrades ECM preferently type I collagen [9].

Urokinase-type plasminogen activator (uPA), lies at the top of the proteolytic cascade of the plasminogen/plasmin system, and acts to generate plasmin from circulating plasminogen by proteolytic cleavage. Plasmin is a broad-spectrum proteinase capable of degrading matrix components directly, and inhibiting deposition of ECM indirectly by activating MMPs secreted in latent inactive forms (in particular pro-MMP1, pro-MMP-3, pro- MMP-9 and pro-MMP-2) [10,11]. Both, MMP-8 and uPA cDNAs have been deviced as therapeutic agents cloned in adenoviral vectors [2,3,9-12]. Their molecular mechanisms have separately been extensively described in different models of experimental cirrhosis.

Because of the regenerative ability and hepatic function are impaired, the remotion of excessive fibrous proteins deposited in the Disse's space and the acceleration of remnant hepatic-mass regeneration, might result in benefit for subjects undergoing liver fibrosis due to the functional re-establishment of the hepatocyte-sinusoid flow exchange. Thus, the goal of this work was to search for the combinatorial effect of gene therapy with adenoviral vectors containing cDNAs for huPA and MMP-8 (Ad-ΔhuPA plus Ad-MMP8) in increasing the survival of cirrhotic animals.

## Methods

### Experimental design

**Figure 1:**
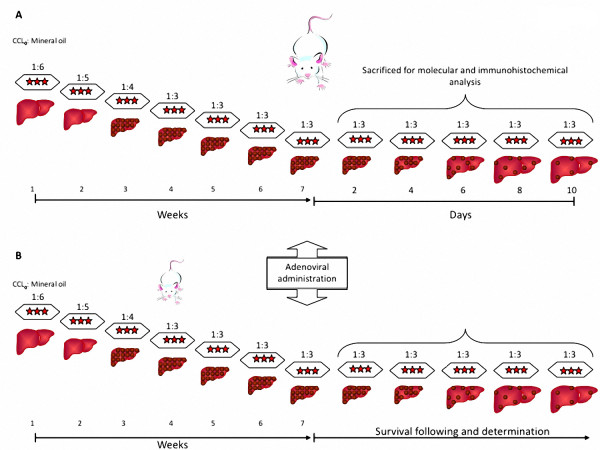
**Experimental design**. Rats were made cirrhotic with different CCl4: mineral oil dilutions during seven weeks. After the animals were treated with the combined gene therapy, they were continuosly injected (three times a week) with the hepatoxin. When indicated, serum and liver extracts were obtained for the determinations specified in Materials and Methods.

Wistar rats, weighing 80 g were made cirrhotic according to Perez-Tamayo R [13]. Then, cirrhotic animals were injected with a dose of combined gene therapy of Ad-ΔhuPA plus Ad-MMP8 (3 × 10^11 ^and 1.5 × 10^11 ^v.p/Kg, respectively) and Ad-β-Gal (4.5 × 10^11^) as irrelevant gene (n = 25 in each experimental group), and were sacrificed at 2, 4, 6, 8 and 10 days after treatment (Figure 1A). At the end of each time period, biological samples (liver, serum, plasma) were obtained for molecular and histological analysis. Four more groups were included for the survival analysis (n = 14) (Figure 1B). It is important to notice that the administration of CCl_4 _was continued up to the end of the experiments in all groups.

### Adenoviral vectors

Worthwhile mentioning, the amount of viral particles of Ad-β-Gal used was identical to the sum of the number of viral particles represented by Ad-huPA and Ad-MMP-8 in order to eliminate experimental artifacts, and to demonstrate that the observed effects were the result of both therapeutic transgenes, and not the result of combining both adenoviruses. The three Ad vectors used were manufactured according to Salgado et al. [14] and Siller et al. [9], under GMPs, GCPs and GLPs.

### Transgene expression and activity assays

Protein extraction and determination were carried out with double- detergent and Bradford metodology, respectivelly. For detection of human MMP8 activity we utilized a comercial kit of Biotrak (Amersham Biosciences, Buckinghamshire, UK). Determination was realized with 40 μg of total proteins of cirrhotic livers-homogenated at 405 nm. For evaluation of MMP-2 and MMP-9 activity we used 40 μg of total protein in each experimental group, according to out previous communication [10]. Human uPA activity was determined using the same methodology, except that gels were covered with casein (1 mg/ml) and plasmingen (1 mg/ml) as substrate.

### Morphometric analysis

For histological study in each experimental group, liver sections were fixed by immersion in 4% paraformaldehyde diluted in phosphate buffered saline (PBS), dehydrated in graded ethylic alcohol and embedded in paraffin. Briefly, sections 6 μm thick were stained with Masson trichromic to determine amount of liver tissue affected by extracellular matrix. Then, by using a computer-assisted automated image analyzer ProPlus (Media Cybernetics, Silver Spring, Md) 20 random fields per slide were analysed and the ratio of connective tissue to the whole liver area was calculated. Results are expresed in mean ± standard deviation.

### Hydroxyproline determination

Liver samples were obtained at the moment of death, and 150 mg of tissue were frozen, weighed and minced into a fine homogenous mixture. Hepatic tissue (1 mg) was hydrolyzed with 2 ml 6N HCl for 12 h at 100°C. Hydroxyproline content of each sample was determined by a colorimetric assay described earlier. Briefly, the reaction was started by adding 1 ml of Chloramine-T solution to 1 ml of sample and 4 ml of Erlich's reactive (dimethylbenzaldehyde acid solution) [15]. After an incubation period of 30 min at room temperature (25-30°C), the optical density (OD) was determined within 30 min at a wavelength of 560 nm. The results were calculated as percent of collagen in wet liver weigh, using hydroxyproline standards (Sigma-Aldrich, Munich, Germany).

### Hepatic functional tests

Blood was drawn from control and experimental cirrhotic animals treated with therapeutical genes at the moment of sacrifice and serum transaminases (ALT and AST), albumin and bilirrubins were determined in an automated Syncron-Cx7 machine at Hospital Civil de Guadalajara.

### Immunohistochemical determinations

Hepatic tissue sections were deparaffinized and rehydrated with xylene and decreasing graded ethanol. Slides were incubated in 3% H_2_O_2 _for 10 min, followed by incubation with polyclonal anti-goat against human uPA (Chemicon International, USA) diluted in PBS (1/400), or incubation with polyclonal antibody anti-goat against human MMP8 (Chemicon International, Temecula, CA, USA) diluted in PBS (1/600). Similarly, the most important proteins implicated in cellular proliferation were determined with either monoclonal anti-mouse PCNA antibody (Sigma Aldrich, Ltd, Dorset, UK) or HGF (Sigma Aldrich, Ltd, Dorset, UK) or c-met (Sigma Aldrich St. Louis, MO) all antibodies were diluted 1/200 in PBS. Marker for HSC activation was determined by a monoclonal anti-mouse antibody against α-SMA (Boehringer Manheim, Germany) diluted 1/50. Primary antibodies were incubated at 4°C overnight, followed by incubation with biotinylated secondary antibodies. Secondary antibodies were complexed individually with avidin-conjugated peroxidase Vectastain ABC-Elite reagent (Vector Laboratories, Burlingame, CA USA) and resulting peroxidase activity was detected with 3,30-diaminobenzidine (DAKO) in sections that were briefly counterstained with hematoxylin. Positive cells were analized in 20 random fields of pericentral, mid-zonal and periportal areas. Cell counting was carried out in a software-automated (Image-Proplus Analyzer, Qwin-Leica, USA).

### Liver profiling gene expression

Gene expression for TGF-β, HGF, c-met and HPRT (constitutive gene) was analyzed by semicuantitative PCR (RT-PCR). RNA extraction was performed by fenol cloroform metodology as reported previously. Retrotranscription was mounted using 2 μg of total RNA and 400 U of M-MLV reverse transcriptase (Gibco, Life Technologies Ltd., Paisley). After that, 2 μl of cDNA plus 48 μl of an solution containing 10× buffer of MgCl_2 _50 mM, 2500 μM dNTPs, 3 μM oligonucleotides, 1U Taq Polimerase (Gibco, Life Technologies Rockville, MD).

The reaction was performed on a Perkin-Elmer DNA Termal Cycler 480, at 94°C (5 mins), 95°C (1 min), 60°C (1 min) and 72°C (1.5 mins). Alignment temperature for HGF was 58°C (1.5 mins). Oligonucleotides utilized are shown in Table 1.

**Table 1:** Primers sequence utilized in the semiquantitative RT-PCR

Gene	Primers
**HPRT**	S 5' TCCCAGCGTCGTGATTAGTG 3' A 3' GGCTTTTCCACTTTCGCTGA 5'
	
**HGF**	S 5' ATGCTCATGGACCCTGGT 3' A 3' GCCTGGCAAGCTTCATTA 5'
	
**c-met**	S 5' CAGTGATGATCTCAATGGGCAAT 3' A 3' AATGCCCTCTTCCTATGACTTC 5'
	
**TGF-β1**	S 5' GCCTCCGCATCCCACCTTTG 3' A 3' GCGGGTGACTTCTTTGGCGT 5'

### Survival analysis

Four additional groups composed by rats with liver fibrosis (n = 14) treated with Ad-β-gal (4.5 × 10^11^vp/kg), Ad-ΔhuPA + Ad-MMP8 (3 and 1.5 × 10^11^vp/kg, respectively), only Ad-MMP-8 (1.5 × 10^11^vp/kg) and only Ad-ΔhuPA (3 × 10^11^vp/kg) were also monitored for their probability of survival.

### Statistical analysis

Data are shown as mean ± SD. Differences between experimental groups and controls were analyzed with ANOVA test. Survival rates were estimated by the Kaplan-Meier method, and differences were analyzed with the log rank test to compare the resulting curves of treatment groups. A probability value of < 0.05 was considered statistically significant (SPSS version 10.0).

## Results

**Figure 2:**
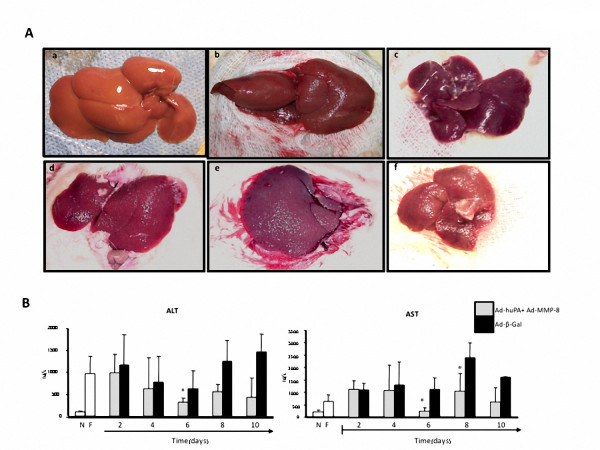
**Macroscopic aspect of liver and functional hepatic tests**. A, a) a representative image a normal rat liver, b and c shows a recovered hepatic smooth texture in animals treated with the combinatorial gene therapy at 8 and 10 days, respectively, as compared with irrelevant gene therapy (e and f), d) shows a control fibrotic liver injected with saline. Histograms in B, show a significative tendency to normal values of hepatic functional tests (ALT and AST).

Reduction of hepatic fibrosis at the end of the experiment (8 and 10 days) resulted in morphological improvement, where a smooth hepatic texture in normal and combined gene therapy-treated cirrhotic rats is conspicuous, as compared with the rough and granular liver surface from Ad-β-Gal-injected animals (Figure 2A). Furthermore, these findings were accompanied by a clear improvement in collateral circulation and gastric varices, suggesting diminished intrahepatic blood pressure in animals injected with Ad-huPA plus Ad-MMP-8 (data no show). Severe accumulation of peritoneal fluid and important clinical manifestations of advanced hepatic cirrhosis were detected in all animals treated with Ad-β-Gal. All cirrhotic animals injected with Ad-huPA plus Ad-MMP8 showed moderate ascites. Functional hepatic tests were notably normalized, after six days of treatment with the therapeutical combination (ALT 43% and AST decreased 75%) compared with cirrhotic animals treated with Ad-β-Gal (Figure 2B).

**Figure 3:**
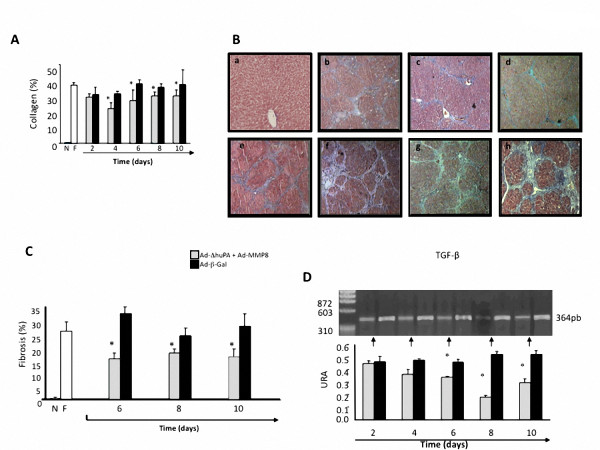
**Fibrosis analysis: histologic and molecular**. A, shows a decrement in collagen content in cirrhotic animals treated with uPA plus MMP-8. B, liver sections Masson's stained in each experimental group, a) a representative histological image of a normal liver rat, in b-d cirrhotic animals treated with combinatorial gene therapy at 6, 8 and 10 days respectively. The histological images of f-h are representative of cirrhotic animals treated with irrelevant vector (Ad-β-Gal) at 6, 8 and 10 days, respectively, e) shows a control fibrotic liver injected with saline. C, Morphometric analysis indicates that animals treated with therapeutically gene therapy has a major recuperation at 6 to 10 days post-treatment (inducing a 55% of regression). D, TGF-β, evaluated by semicuantitative RT-PCR, showed a major decrement in mRNA at 8 days after treatment with Ad-uPA plus Ad-MMP-8 as compared with irrelevant control. N, normal; F, fibrotic.

Collagen content, as measured by the fibrosis index, demonstrated that only cirrhotic animals treated with therapeutical combination showed a significative regression of fibrosis. Hydroxyproline content decreased in cirrhotic animals treated with Ad-huPA plus Ad-MMP8 compared with the group administered with Ad-β-Gal. As shown in Figure 3A, the most important regression of fibrosis was at 4 days after administration of therapeutical vectors. To corroborate these findings, we realized histo-morphometric analysis that showed less ECM-accumulation in the animals treated with Ad-huPA plus Ad-MMP-8 at the 6^th ^day (50%), (Figure 3B and 3C). Gene expression of the most important profibrogenic-cytokine (TGF-β) was significantly diminished in this same group of animals treated with therapeutic adenoviral vectors (60% of decrement at 8^th ^day), (Figure 3D).

**Figure 4:**
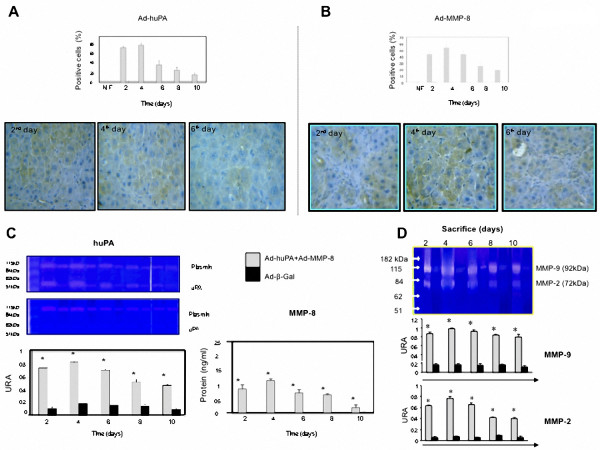
**Human transgenes detection and fibrinolytic activities**. Panels A and B show uPA and MMP-8 proteins in cirrhotic animals, both control and experimental (2^nd ^through 10^th ^days) detected by immunohistochemistry, indicating percentage of positive cells. C, transgenes activity, shows major activity of both trangenes (uPA and MMP-8) at 4 days after treatment. D, both enzymes (MM-2 and MM-9) increment their functional activities only in the cirrhotic animals treated with Ad-uPA plus Ad-MMP-8 and the maximum activity was at 4 days post-treatment.

Immunohistochemical determination of human trangenes (huPA and MMP-8) was performed in each experimental group. A significative increment of the corresponding proteins was observed at 4 day after therapeutical vectors injection to cirrhotic animals as compared with the group of rats treated with irrelevant control, demonstrating an efficient transduction of adenoviral vectors, (Figure 4A and 4B). Then, we proceeded to determine the actual biological activity shown by human uPA that had a molecular migration at 54 kDa. Similarly, the major activity of transgenes was observed at 4 days after administration of vectors (Figure 4C). The activity of MMP2 and MMP9 significantly increased at 4 day after treatment in experimental group treated with Ad-huPA plus Ad-MMP-8 and correlated with the presence and activity of human transgenes transduced by Ad vectors (huPA and MMP-8). Figure 4D, clearly shows activity for both matrix metalloproteinase's (MMP-2; 72 kDa and MMP-9; 92 kDa).

**Figure 5:**
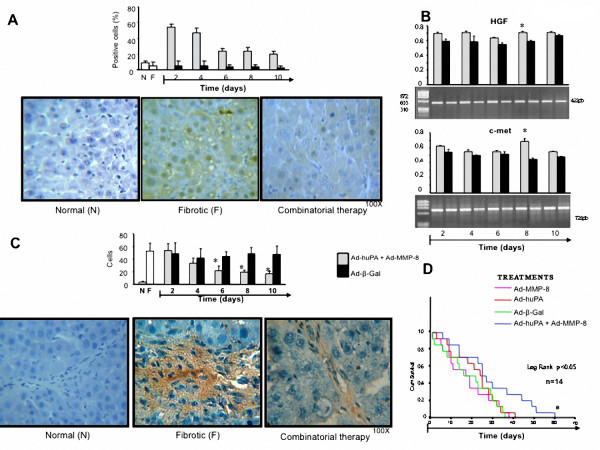
**Cellular proliferation and survival**. A, PCNA protein (regeneration index) shows increment only with the treatment of combinatorial gene therapy at 2-4 days post treatment. B, gene expression of HGF and c-met determined by semiquantitative RT-PCR that indicate a light increment of both genes on the animals treated with Ad-uPA plus Ad-MMP-8 to respect Ad-β-Gal. C, activated HSC (α-SMA^+^) show significant decrement after 2 days of treatment with therapeutically gene therapy. D, Survival analysis (Kaplan Meier) of cirrhotic animals in each experimental group. Increment of survival (60 days) in the animals receiving the combined gene therapy is noticeable.

Hepatic cells proliferation determined by anti-PCNA immunohistochemical revealed an increment (50%) in the number of hepatocytes of animals receiving combinatorial gene therapy during the first four days of treatment (at 2 and 4 days of sacrifice). Number of PCNA positive cells began to decrease afterwards (Figure 5A). Figure 5B shows gene expression for HGF and its cognate receptor c-met determined by semiquantitative RT-PCR. Only cirrhotic animals treated with AdhuPA plus AdMMP-8 shown decrement of number of activated-HSC (α-SMA^+^) as of the fourth day (Figure 5C).

Finally, the most relevant piece of data shown here is the determination of survival, which was significative only in cirrhotic animals that recover after the treatment with the combined therapeutic vectors (about 30% of recovery). The surviving animals reached 60 days after combinatorial therapy as compared with only 35 to 40 days displayed by animals injected either with irrelevant vectors or mono therapy, (Figure 5D).

## Discussion

Liver cirrhosis is becoming an increasing health problem; patients with liver cirrhosis have a high mortality, not just from cirrhosis-related causes, but also from other causes. This observation indicates that many patients with cirrhosis have other chronic diseases, yet the prognostic impact of co-morbidities has not been examined [16]. Common causes of liver cirrhosis include hepatitis B, hepatitis C, alcohol abuse as well as non-alcoholic steato-hepatitis and hereditary metabolic defects. Liver cirrhosis has a considerable impact on surgical practice [17].

Liver fibrosis refers to the accumulation of interstitial ECM (scar) after chronic hepatic injury. Septum formation and rings of scar that surround nodules of hepatocytes characterize cirrhosis, the end-stage of progressive fibrosis. These structural alterations that repel in the architecture of liver are characterized by an alteration in the wound healing of ECM, conversion of HSC into activated myofibroblast-type cells and hepatocyte proliferative arrest [6,11]. These alterations have been derived of genetic overexpression or down-regulation in the activity of enzymes participating in the normal mechanisms of ECM degradation (metalloproteases) Also, can be caused by growth factors (TGF-β or PDGF) influencing HSC activation characterized by the acquisition of a proliferative, contractile, migratory, fibrogenic and inflammatory phenotype [6]. To experimentally reproduce these alterations, several approaches for induction of fibrosis have been described. Of these, CCl_4 _chronic intoxication in rats and mice is probably the most widely studied. In addition, CCl_4 _model is the best characterized with respect to histological, biochemical, cellular, and molecular changes associated with the development of human hepatic fibrosis. CCl_4 _given intraperitoneally induces hepatocyte necrosis and apoptosis with associated HSC activation and tissue fibrosis. The ongoing treatment with CCl_4 _can be used to induce hepatic fibrosis (4 weeks), cirrhosis (8 weeks) and advanced micronodular cirrhosis (12 weeks) [5]. In this experimental work the results showed a timing onset of hepatic fibrosis characterized by distortion of normal architecture of liver, with extensive fibrous proteins (scarring) that create fibrotic-bridges between contiguos hepatic lobules, HSCs activation, decrement in metalloproteases function, increment of TGF-β1 gene expression and fibrillary proteins. Reversal of these processes (histological, biochemical, cellular, and molecular) has become the focus in the treatment of liver fibrosis.

With the development of gene therapy for various liver diseases, intensive efforts have been made to design gene therapy strategies aimed at blocking any of the fibrogenic pathways, regulate the fibrinolytic homeostasis and re-establishment of organic functional activity [12,18].

As uPA was able to induce ECM-degradation, we determined if this fibrosis regression was induced by an increase in MMP-2 and MMP9 activity, which exacerbate type I collagen degradation. We also demonstrated that infusion with uPA gene to the animal model produced increased expression of uPA protein, resulting in significant attenuation of fibrosis.

Stimulation of hepatocyte regeneration is one of the essential strategies for the treatment of hepatic fibrosis. HGF is considered to be the strongest hepatocyte proliferative agent to date and is able to exhibit a plethora of effects in hepatic fibrosis. HGF could stimulate hepatocyte mitosis, inhibit hepatocyte apoptosis, and suppress the expression of TGF-β1, resulting in inhibition of proliferation and activation of HSC in the fibrotic liver [19]. It could be inferred that HGF is able to stimulate hepatocyte regeneration and remodel the deranged cirrhotic tissue as well, offering the substantial potential for gene therapy of liver cirrhosis [20]. The synergistic antifibrotic effect on hepatic fibrosis was attributed to some potential mechanisms. First, uPA increases gene expression of HGF and had a proliferative effect on hepatocytes [14,20]. Overexpression of HGF could also result in suppression of TGF-β1 in the hepatic wound-healing response.

At present, combinational gene delivery seemed to be one of the most important developments in gene therapy. Yang and his colleagues reported that combinational gene therapy using IL-12, pro-IL-18, and IL-1β converting enzyme (ICE) cDNA expression vectors simultaneously delivered via a gene gun could significantly augment anti-tumor effects by generating increased levels of bioactive IL-18 and consequently IFN-γ. Similarly, Lyn *et al*. have shown the therapeutic effect of combination of uPA plus HGF on experimental liver fibrosis. They transferred HGF gene into primary cultured hepatocytes and uPA gene to hepatic stellate cell (HSC) to investigate the effect on the biological character of cells. Transfection of exogenous HGF gene stimulated hepatocyte proliferation. Human uPA gene decreased the amount of type I and III collagens accompanied with increased expression of matrix metalloproteinase-2 *in vitro*. *In vivo*, the area of ECM in the fibrotic liver decreased to 72% in Ad-HGF-treated rats, 64% in the Ad-uPA-treated group, and 51% in bi-genes transfection. Moreover, immunohistochemical staining of collagen types I and III revealed that combinational genes delivery exerted more effect on reversal of hepatic fibrosis than mono-gene transfection. This study indicated that simultaneous delivery genes could confer synergistic effect on hepatic fibrosis [11].

According to our study, the most interesting finding was that the combinational delivery of uPA and MMP-8 genes was more effective than mono-gene therapy in reversal of fibrosis and is in agreement with Lyn *et al*. respect to sinergystic effect of fibrosis regression (Figure 3), increment of functional activity of MMP2 and MMP-9 (Figure 4D) involved in the degradation of the excessive collagens deposition, in a persistent hepatic fibrosis animal model intoxicated continuosly with CCL_4_.

In fact, even if the liver has an enormous functional reserve and a unique regenerative capacity, cirrhotic liver regenerates less actively than normal liver. The discovery of agents that could sustain cirrhotic liver regeneration would thus have important clinical implications. Osawa *et al*. reported that HGF plus truncated type II transforming growth factor-β receptor (TβRII), stimulates liver regeneration, accelerates restoration of hepatic function, and prevents progression of liver fibrosis. Tiberio et al. have shown incremented the survival in decompensated cirrotic animals treated with human-IL-6 recombinant [17].

Our results provide evidence that uPA plus MMP-8 treatment reduces mortality. We speculate that the increased survival of cirrhotic rats may in part result from uPA-mediated enhancement and acceleration of liver regeneration that we directly demonstrated by increments in HGF, c-met and PCNA gene expression in rats affected by cirrosis and liver function, which included serum levels of ALT and AST, improved significantly in therapeutic gene therapy-treated rats compared with all other groups.

Indeed, accelerated recovery of the liver mass, and significant increments of ECM-degradation contributed to survival of cirrhotic animals. Combinational delivery of uPA plus MMP-8 genes would be reflected in a significative increment in survival time (Figure 5D), which had advantages over the treatment with either Ad-huPA or Ad-MMP-8 alone. Our findings suggested that simultaneous delivery of two or multiple and functional therapeutic genes, can provide a new biotechnological weapons for the treatment of hepatic fibrosis.

In general, our results suggest that the combination of uPA and MMP-8 gene therapy may increase the possibility of survival in cirrhotic animals by improving fibrosis, function, and hepatocyte regeneration.

## Competing interests

The authors declare that they have no competing interests.

## Authors' contributions

FJGG, AASF, MDSG and JFMV participated in the design of the study. JSAB drafted the manuscript, conceived the study, and participated in its design and coordination. All authors read and approved the final manuscript.
